# Supplementary material for: Identification of photoperiod-induced specific miRNAs in the adrenal glands of Sunite sheep (Ovis aries)
Source: Front Vet Sci. 2022 Jul 22;9:888207. doi: 10.3389/fvets.2022.888207 (PMC9354845; doi:10.3389/fvets.2022.888207)
Supplement: Supplementary file 1 [file Data_Sheet_1.ZIP › Supplementary file/miRDeep2 file.zip!/miRDeep2 file/Know_Structure/SPLP42A2/oar-mir-376e.pdf]

miRBase precursor : oar-mir-376e
Total read count : 930
oar-miR-376e-5p read count : 192
oar-miR-376e-3p read count : 546
oar-miR-376b-5p read count : 192
remaining reads : 0

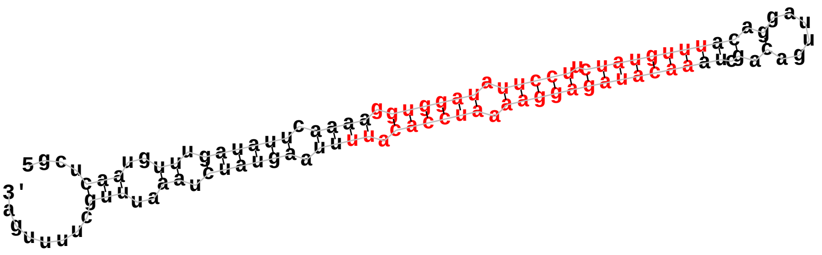

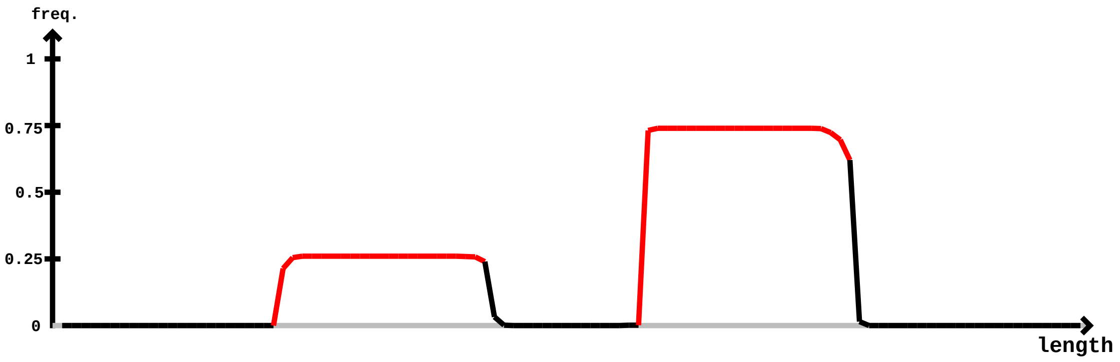


```
                                                                oar-miR-376e-3p
                      oar-miR-376b-5p
                      oar-miR-376e-5p
5'- gcucaauguuugauauucaaaagguggauauuccuucuauguuuacaggauugacagcuaaacauagaggaaaauccacauuuuaaguaucuaaaauuugcuuuuga   -3'    exp
    ((.....((((((((((.((((.((((((.(((((.((((((((((.(......).).)))))))))))))).)))))).)))).)))))).))))..))......       reads     mm      sample
    ......................gguggauauuccuucuaug..................................................................       1         0       seq
    ......................gguggauauuccuucuaugu.................................................................       1         0       seq
    ......................gguggauauuccuucuauguu................................................................       13        0       seq
    ......................gguggauauuccuucuauguuu...............................................................       125       0       seq
    ......................gguggauauuccuucuauguuua..............................................................       18        0       seq
    .......................guggauauuccuucuauguuu...............................................................       26        0       seq
    .......................guggauauuccuucuauguuua..............................................................       4         0       seq
    ........................uggauauuccuucuauguuu...............................................................       2         0       seq
    ........................uggauauuccuucuauguuua..............................................................       1         0       seq
    ........................uggauauuccuucuauguuuac.............................................................       1         0       seq
    ..........................................................uaaacauagaggaaaauccaca...........................       1         0       seq
    ............................................................aacauagaggaaaaucca.............................       1         0       seq
    ............................................................aacauagaggaaaauccac............................       11        0       seq
    ............................................................aacauagaggaaaauccaca...........................       19        0       seq
    ............................................................aacauagaggaaaauccacau..........................       56        0       seq
    ............................................................aacauagaggaaaauccacauu.........................       444       0       seq
    ............................................................aacauagaggaaaauccacauuu........................       8         0       seq
    .............................................................acauagaggaaaauccacauu.........................       3         0       seq
    .............................................................acauagaggaaaauccacauuu........................       3         0       seq
```
